# Washed microbiota transplantation improves patients with high blood glucose in South China

**DOI:** 10.3389/fendo.2022.985636

**Published:** 2022-09-23

**Authors:** Lei Wu, Man-Qing Li, Ya-Ting Xie, Qing Zhang, Xin-Jian Lu, Tao Liu, Wen-Ying Lin, Jia-Ting Xu, Qing-Ping Wu, Xing-Xiang He

**Affiliations:** ^1^ Department of Gastroenterology, Research Center for Engineering Techniques of Microbiota-Targeted Therapies of Guangdong Province, The First Affiliated Hospital of Guangdong Pharmaceutical University, Guangzhou, China; ^2^ Guangdong Provincial Key Laboratory of Microbial Safety and Health, State Key Laboratory of Applied Microbiology Southern China, Institute of Microbiology, Guangdong Academy of Sciences, Guangzhou, China; ^3^ School of Biology and Biological Engineering, South China University of Technology, Guangzhou, China

**Keywords:** fecal microbiota transplantation, washed microbiota transplantation (WMT), abnormal blood glucose metabolism, high blood glucose, fasting blood glucose

## Abstract

**Background and Aims:**

Although fecal microbiota transplantation (FMT) from healthy donors has been shown to have hypoglycemic effects in animal models of diabetes, its clinical impact in patients with abnormal blood glucose metabolism is unclear, especially in southern Chinese populations. The aim of this study was to investigate the feasibility and efficacy of washed microbiota transplantation (WMT) in the treatment of abnormal blood glucose metabolism in a population in southern China.

**Methods:**

The clinical data of patients with different indications who received 1-3 treatments of WMT were retrospectively collected. The changes of blood glucose, blood lipids, blood pressure, liver function and blood routine before and after WMT were compared, such as fasting blood glucose (FBG), glycosylated hemoglobin (HbA1c), total cholesterol (TC), triglyceride (TG), systolic blood pressure (SBP), white blood cells (WBC), lymphocytes (LY) and platelets (PLT), etc.

**Results:**

A total of 195 patients were included in the First Affiliated Hospital of Guangdong Pharmaceutical University, including 20 patients with high blood glucose and 175 patients with normal blood glucose. WMT has a significant effect in reducing short term blood glucose level (FBG) in patients with high blood glucose (p < 0.05). The fasting blood glucose (FBG) of 72.22% of patients with high blood glucose decreased to normal in a short term (about 1 month) (p < 0.001); In the medium term (about 2 months), there was a significant hypolipidemic (TG) (p = 0.043) effect, long term (about 6 months) significant blood pressure lowering (SBP, p = 0.048) effect. Overall, WMT significantly reduced the risk of high risk classes of Atherosclerotic Cardiovascular Disease (ASCVD) in the short term (p = 0.029) and medium term (p = 0.050).

**Conclusion:**

WMT can significantly improve blood glucose in patients with high blood glucose, and there is no long-term elevated risk of blood glucose and ASCVD. FBG levels were significantly reduced in both the short and medium term in patients with high blood glucose treated with WMT. Therefore, the regulation of gut microbiota by WMT may provide a new clinical approach for the treatment of abnormal blood glucose metabolism.

## Introduction

Diabetes mellitus, a chronic disease characterized by relative or absolute insulin deficiency leading to hyperglycemia, is one of the largest global public health problems. The prevalence of diabetes has continued to increase in most developed and developing countries in recent decades (1, 2). To date, the International Diabetes Federation (IDF) estimates that approximately 451 million adults (aged 18-99) were living with diabetes worldwide in 2017, and this is expected to increase to 693 million by 2045 if effective prevention methods are not taken (3). In China, the prevalence of diabetes is still rising, with the prevalence increased rapidly to 10.9% - 12.8% from 2010 to 2018. Diabetes awareness (36.5%), treatment (32.2%) and control (49.2%) rates have improved but remain low (4). The prevalence of diabetes was significantly higher in urban areas than in rural areas (12.0% vs 8.9%), and higher in men than in women (11.1% vs 9.6%) (5, 6). Type 2 diabetes mellitus (T2DM) accounts for more than 90% of the diabetic population, and the prevalence of diabetes in the elderly over 60 years old is close to or exceeds 20% (7). At the same time, diabetes is one of the top ten causes of death in the world, and people with diabetes mainly die of diabetes-related complications rather than diabetes itself (8). A large number of these premature deaths can potentially be prevented through prevention or early detection, and improved management of diabetes and these complications.

T2DM patients often have one or more components of metabolic syndrome, such as hypertension, dyslipidemia, obesity, etc., which significantly increase the risk, progression speed and harm of T2DM complications. Therefore, a scientific and reasonable T2DM treatment strategy should be comprehensive, including the control of blood glucose, blood pressure, blood lipids, and body weight, antiplatelet therapy and lifestyle improvement measures. These treatment strategies are mainly lifestyle intervention and drug therapy. A variety of drugs are used to treat T2DM and can be divided into five main categories: drugs that stimulate insulin production by beta cells (sulfonylureas), improve insulin action (thiazolidinediones), delay carbohydrate uptake in the gut (alpha-glucosidase inhibitors), reduce hepatic glucose production (metformin) or target the glucagon-like peptide (GLP-1) axis (GLP-1 receptor agonists or DPP-4 inhibitors) (9). However, long term use of drug therapy can have significant side effects. Therefore, it is of great significance to comprehensively analyze the related factors of diabetes and find a treatment method with less side effects.

Gut microbiota has emerged as an important factor associated with human disease (10, 11). Numerous studies have demonstrated changes in the gut microbiota of T2DM patients by comparing the gut microbiota of T2DM patients with healthy individuals (12, 13). Dietary fiber selectively promotes gut bacteria to alleviate T2DM (14), suggesting that gut microbiota plays an important role in T2DM. In animal models of T2DM, Fecal microbiota transplantation (FMT) can improve HOMA-IR and insulin sensitivity (15), and repair damaged pancreatic islets (16), providing a potential strategy for the treatment of T2DM (17). FMT is a new treatment approach that uses healthy microbial profiles to replace the patient’s own microbiota (18). FMT is of increasing interest (19) and have been successfully used to treat a variety of human diseases, such as inflammatory bowel disease (20), obesity (21), as well as metabolic syndrome (22) and functional bowel disease (23, 24). Whether FMT can improve abnormal blood glucose metabolism is a topic to be explored in clinical medicine.

Washed microbiota transplantation (WMT) is safe, quality-controlled (25), and effective (26) for the treatment of gut microbiota disorders. We attempted to investigate whether WMT could improve blood glucose metabolism in patients undergoing WMT. We hypothesized that WMT could safely and consistently affect patients across a variety of indications, improving abnormal blood glucose metabolism without side effects. Therefore, we conducted a retrospective trial to collect medical data from patients with abnormal blood glucose metabolism treated with WMT.

## Materials and methods

### Patients

This study was approved by the Ethics Committee of the First Affiliated Hospital of Guangdong Pharmaceutical University, Guangzhou, China according to the Declaration of Helsinki (no. 2017-98). Written informed consent was obtained and reviewed from all patients. This study included patients who completed 2-4 courses of WMT in our hospital from December 7, 2016 to April 30, 2022. Inclusion criteria: older than 18 years, informed consent, and those who received WMT. Exclusion criteria: pregnant women, patients taking antibiotics, hormones and probiotics during the first 3 months of WMT and during transplantation, and patients taking hypoglycemic drugs after WMT. In the end, a total of 195 people met the requirements.

### WMT procedure

The procedure of WMT was in accordance with the Nanjing Consensus on the Methodology of Washed Microbiota Transplantation (26). All healthy fecal donors between the ages of 18 and 25 undergo rigorous consultation, psychological and physical examination, biochemical testing and infectious disease screening. To prepare the washed microbiota, each 100 g of feces and 500 mL of 0.9% saline was used to prepare a homogeneous fecal suspension. The fecal suspension was then subjected to microfiltration (to remove fecal particles, parasite eggs, and fungi) using a smart microbial separation system (GenFMTer; FMT Medical, Nanjing, China). After microfiltration, the fecal supernatant was centrifuged at 1100 × g for 3 min at room temperature. Then, the supernatant obtained after centrifugation was discarded. The microbiota pellet was resuspended in saline, then the microbiota pellet was centrifuged and resuspended 3 times. In the final resuspension, 100 mL of saline was added to the microbiota pellet obtained from 100 g of feces (25). Then, according to each patient’s physical condition and wishes, the fecal suspension was injected into the patient through a nasojejunal tube (upper gastrointestinal tract) or a colonic transendoscopic enteral tube (lower gastrointestinal tract). A WMT course of 3 days, once a day, once a 120ml (27). The results of blood tests and other tests before the first course of treatment are the baseline values, and relevant indicators will be obtained before each subsequent course of treatment. According to the standard treatment time of WMT, short term: about 1 month interval from the first course of WMT; medium term: about 2 months interval from the first course of WMT; long term: about 6 months interval from the first course of WMT. All patients received at least 2 courses of WMT and completed follow-up.

### Data collection

The medical records of the patients before treatment (baseline value), short term efficacy, medium term efficacy, and long-term efficacy were collected. Data included age, sex, body mass index (BMI), blood glucose at admission, blood pressure, disease or indication for WMT, and laboratory test results. Blood glucose indicators, namely fasting blood glucose (FBG), glycosylated hemoglobin (HbA1c). Insulin indicators, namely fasting insulin (FI), homeostasis model assessment of insulin resistance (HOMA-IR). Blood lipid indicators, namely total cholesterol (TC), triglyceride (TG), low density lipoprotein cholesterol (LDL-c), high density lipoprotein cholesterol (HDL-c), apolipoprotein B (ApoB), non-high density lipoprotein (non-HDL-c), lipoprotein (LIP). Blood pressure indicators, namely systolic blood pressure (SBP) on admission, diastolic blood pressure (DBP) on admission. Liver function indicators, namely alanine aminotransferase (ALT), aspartate aminotransferase (AST), serum albumin (ALB), total protein (TP), Albumin/globulin ratio (A/G), leucine aminopeptidase (LAP), glutamyl transpeptidase (GT), glutathione reductase (GR), Direct bilirubin (DBIL), indirect bilirubin (IBIL), total bilirubin (TBIL). Blood routine, that is, red blood cell count (RBC), hemoglobin concentration (HGB), white blood cell count (WBC), lymphocyte count (LY), platelet count (PLT), etc. HOMA-IR values were calculated as previously described (28). According to the Chinese Guidelines for the Prevention and Treatment of Type 2 Diabetes (2020 Edition), patients with high blood glucose were diagnosed (4), and all eligible patients were divided into 2 groups: high blood glucose group, 6.1mmol/L≤FBG or 6.1%<HbA1c and no hypoglycemic drugs, or FBG ≥7.0mmol/L was diagnosed as diabetic but without hypoglycemic drugs. Diagnosed with diabetes and the use of hypoglycemic drugs were excluded. Normal blood glucose group, 3.9mmol/L≤FBG<6.1mmol/L. Hospital-defined hyperlipidemia (TC ≥ 6.2 mmol/L or TG ≥ 2.3 mmol/L or LDL-c ≥ 4.1 mmol/L), hypolipidemia (TC < 3.6 mmol/L or TG < 0.33 mmol/L or LDL-c < 2.07 mmol/L or HDL < 0.91 mmol/L). Hypertension was defined as SBP ≥140 mmHg and/or DBP ≥90 mmHg (29). With reference to the Chinese cardiovascular disease prevention guidelines (2017 edition) (30), ASCVD risk stratification was performed according to baseline and blood lipid status: Those who meet one of the following conditions were directly classified as high-risk groups (1): Diabetes (age ≥ 40 years old) (2). Individuals with extremely high levels of a single risk factor, including: ① LDL-c ≥ 4.9 mmol/L (190 mg/dl) or TC ≥ 7.2 mmol/L (280 mg/dl); ② Grade 3 hypertension; ③ Heavy smoking (≥ 30 sticks/d). According to the 10-year average risk of ASCVD in different combinations, it was defined as low risk, intermediate risk and high risk according to < 5%, 5%-9% and ≥ 10%, respectively. Obese (≥ 28.0), overweight (24.0-28.0), normal weight (18.5-24.0), and underweight (< 18.5) were defined according to BMI (kg/m^2^) (31). Definition of serious adverse events (AEs): increased frequency of defecation, fever, abdominal pain, flatulence, hematochezia, vomiturition, bloating and herpes zoster, etc (25). All patients were divided into 2 groups according to the above definitions. After all patients received 1-3 WMT treatments and completed follow-up, statistical analysis and evaluation of blood glucose, blood lipids, blood pressure, liver function and blood routine results were performed.

### Statistical analysis

Statistical analysis was performed using SPSS 22.0 (IBM Corp., Armonk, NY, USA) and Prism 8 (GraphPad, San Diego, CA, USA). Results are expressed as frequencies and percentages for categorical variables, mean and standard deviation for continuous variables with a normal distribution. Categorical variables were analyzed using chi-square or Fisher’s exact test. For comparison of continuous variables between two independent groups, an unpaired Student’s-t test (normally distributed variables) can be used. Paired data were compared using paired Student’s-t test (normally distributed variables). Two-tailed p-values ≤ 0.05 were considered statistically significant.

## Results

### Clinical characteristics of patients who underwent WMT

**Figure 1:**
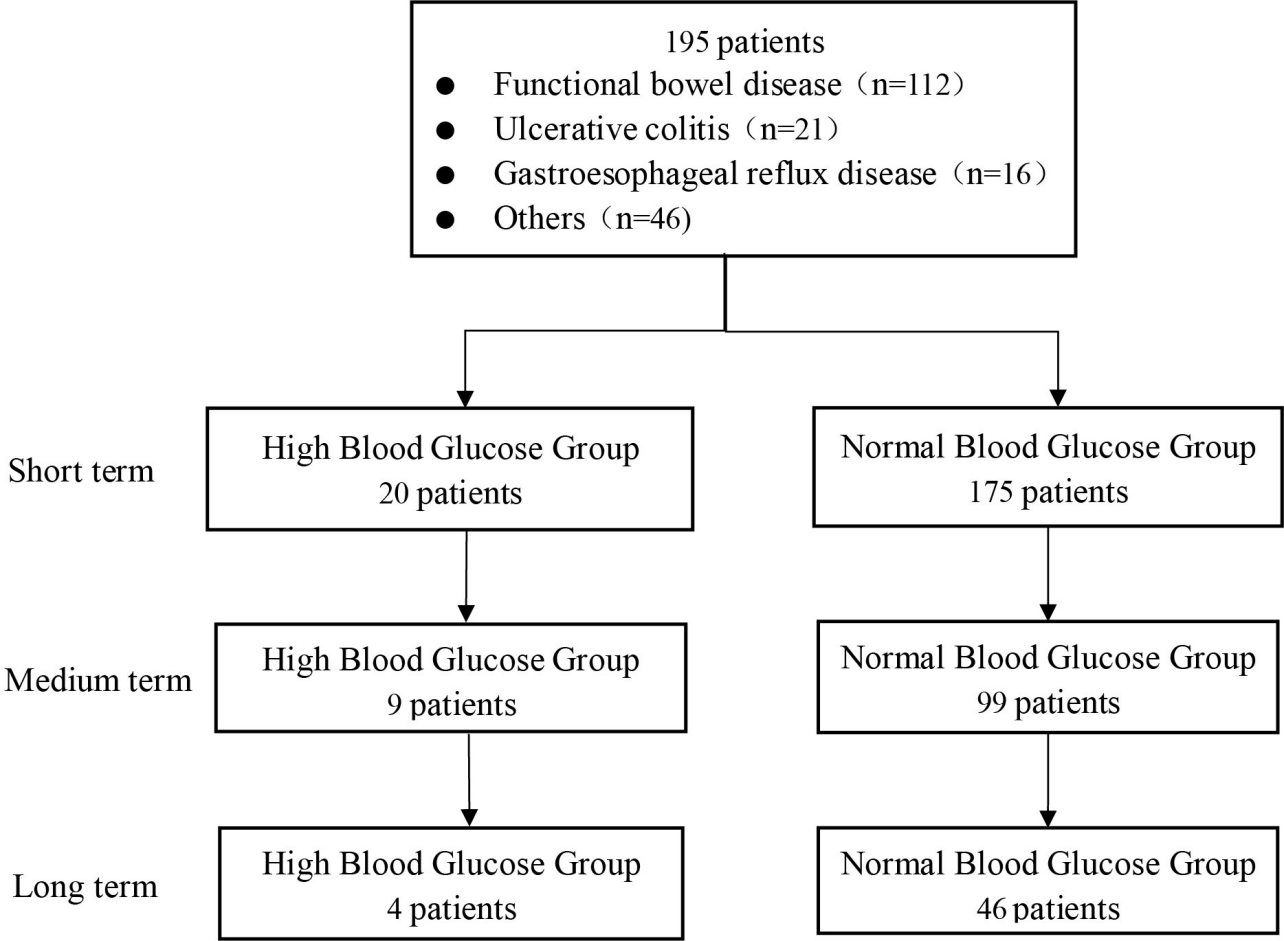
Flow chart of this study.

WMT was completed in the First Affiliated Hospital of Guangdong Pharmaceutical University from December 2016 to April 2022. A total of 195 patients met the inclusion criteria (20 in the high blood glucose group and 175 in the normal blood glucose group). Among them, 98 (50.26%) were male and 97 (49.74%) were female, with an average age of 52.59 ± 14.36 years (**Figure 1** **)**. **Table 1** shows the main six disease characteristics of patients undergoing WMT, which are functional bowel disease (n=112, 57.44%, including irritable bowel syndrome, functional constipation), ulcerative colitis (n=21, 10.77%), gastroesophageal reflux disease (n=16, 8.21%), gouty arthritis (n=7, 3.59%), atopic dermatitis (n=6, 3.08%), chemotherapy-related diarrhea (n=6, 3.08%), hyperlipidemia (n=6, 3.08%). Due to the different compliance of patients, WMT treatment may not be completed on schedule. In this study, the time interval of WMT in the enrolled patients was counted, and the number of days was expressed as the median (25%-75%). The blood test results of the patients before the first course of treatment were the baseline values, and the interval between 3 treatments was 35 days (32-42 days) in the short term, 77 days (67-97.75 days) in the medium term, and 183 days (145-202.25 days) in the long term.

**Table 1:** The main diagnoses of patients receiving washed microbiota transplantation.

Primary cause of WMT	Number (n)	Percentage (%)
Functional bowel disease	112	57.44%
Ulcerative colitis	21	10.77%
Gastroesophageal reflux disease	16	8.21%
Gouty arthritis	7	3.59%
Atopic dermatitis	6	3.08%
Chemotherapy-associated diarrhea	6	3.08%
Hyperlipidemia	6	3.08%
Radiation enteritis	5	2.56%
Hyperuricemia	3	1.54%
Hepatitis B cirrhosis	1	0.51%
Hyperlipidemic pancreatitis	1	0.51%
Functional dysphagia	1	0.51%
Senile tremor	1	0.51%
Epilepsy	1	0.51%
Duodenal stasis	1	0.51%
Crohn’s disease	1	0.51%
Chronic urticaria	1	0.51%
Depression	1	0.51%
Parkinson’s syndrome	1	0.51%
Bipolar disorder	1	0.51%
Psoriasis vulgaris	1	0.51%
Perianal eczema	1	0.51%
Total	195	100%

The demographic and clinical characteristics of high blood glucose group versus normal blood glucose group patients are compared in **Table 2**. Due to different compliance, not all patients have complete data, so the number of patients in each group is different for each indicator. There was no significant difference in age, gender ratio, and BMI between high blood glucose group and normal blood glucose group, indicating that the basic conditions of the study population were not significantly different, reducing the confounding factors of this study. The mean FBG of the high blood glucose group was 6.6 ± 1.67mmol/L higher than the definition in Materials and methods, and the mean FBG of the normal blood glucose group was 4.62 ± 0.48mmol/L. There was a significant difference in FBG between the two groups. The average TG in the high blood glucose group was 3.33 ± 4.75mmol/L, which was higher than the definition of hyperlipidemia, and that in the normal blood glucose group was 1.2 ± 0.78mmol/L. There was a significant difference in TG between the two groups. This indicated that the high blood glucose group was accompanied by symptoms of hyperlipidemia in addition to the higher FBG. HbA1c (6.02 ± 0.78 vs 5.41 ± 0.30%), FI (14.71 ± 10.86 vs 8.12 ± 4.93 μU/mL), HOMA-IR (4.26 ± 3.3 vs 1.7 ± 1.1), non-HDL-c (4.15 ± 1.65 vs 3.4 ± 0.98 mmol/L), AST (26.1 ± 11.53 vs 20.98 ± 7.12 U/L), LAP (33.2 ± 8.93 vs 29.71 ± 4.8 U/L), GT (60.34 ± 80.04 vs 23.88 ± 14.04U/L), DBIL (5.82 ± 10.41 vs 4.25 ± 1.85 μmol/L), TBIL (15.08 ± 24.8 vs 13.16 ± 5.62μmol/L), WBC (7.58 ± 3.22 vs 5.66 ± 1.69 10^9^/L), PLT (256.6 ± 102.96 vs 224.36 ± 56.79 10^9^/L), these indicators were significantly higher in the high blood glucose group than in the normal blood glucose group. There were only 10 AEs (1.82%) among the 548 times undergoing WMT treatment, mainly diarrhea (3 patients, 0.55%), sore throat (2 patients, 0.36%), and anal pain (2 patients, 0.36%)), nausea (1 patient, 0.18%), dizziness (1 patient, 0.18%), joint soreness (1 patient, 0.18%). There were no serious AEs, and it resolved itself within a few days.

**Table 2:** Demographics and clinical characteristics of patients at baseline.

	High blood glucose group (20)	Normal blood glucose group (175)	p-Value
Age (year)	54.8 ± 13.42 (n = 20)	50.38 ± 15.29 (n = 175)	0.275
Male n (%)	48.57	65	0.164
BMI (kg/m^2^)	24.75 ± 5.56 (n = 17)	22.07 ± 3.94 (n = 174)	0.423
FBG (mmol/L)	6.6 ± 1.67 (n = 20)	4.62 ± 0.48 (n = 175)	<0.001
HbA1c (%)	6.02 ± 0.78 (n = 11)	5.41 ± 0.30 (n=12)	0.007
FI (μU/mL)	14.71 ± 10.86 (n = 11)	8.12 ± 4.93 (n =94)	<0.001
HOMA-IR	4.26 ± 3.3 (n = 11)	1.7 ± 1.1 (n = 94)	<0.001
TC (mmol/L)	5.24 ± 1.45 (n = 17)	4.73 ± 1.03 (n = 157)	0.206
TG (mmol/L)	3.33 ± 4.75 (n = 17)	1.2 ± 0.78 (n = 157)	<0.001
LDL-c (mmol/L)	2.82 ± 0.94 (n = 17)	2.87 ± 0.91 (n = 157)	0.511
HDL-c (mmol/L)	1.09 ± 0.3 (n = 17)	1.32 ± 0.32 (n = 157)	0.827
ApoB (g/L)	1.04 ± 0.31 (n = 17)	0.91 ± 0.23 (n = 157)	0.741
non-HDL-c (mmol/L)	4.15 ± 1.65 (n = 17)	3.4 ± 0.98 (n = 157)	0.029
LIP (mmol/L)	193.34 ± 228.31 (n = 8)	160.31 ± 235.92 (n = 49)	0.366
SBP (mmHg)	130.2 ± 19.87 (n = 20)	121.5 ± 13.08 (n = 175)	0.130
DBP (mmHg)	78.75 ± 10.84 (n = 20)	77.11 ± 9.7 (n = 175)	0.592
ALT (U/L)	23.55 ± 13.14 (n = 20)	20.54 ± 16.57 (n=174)	0.817
AST (U/L)	26.1 ± 11.53 (n = 20)	20.98 ± 7.12 (n = 174)	0.015
ALB (g/L)	38.01 ± 6.29 (n = 20)	40.7 ± 4.32 (n=174)	0.307
TP (g/L)	67.87 ± 5.1 (n = 20)	68.3 ± 6.01 (n = 174)	0.219
A/G	1.33 ± 0.36 (n = 20)	1.53 ± 0.33 (n = 174)	0.972
LAP (U/L)	33.2 ± 8.93 (n = 12)	29.71 ± 4.8 (n = 133)	0.006
GT (U/L)	60.34 ± 80.04 (n = 11)	23.88 ± 14.04 (n = 142)	<0.001
GR (U/L)	59.66 ± 16.49 (n = 17)	56.51 ± 12.7 (n = 128)	0.105
DBIL (μmol/L)	5.82 ± 10.41 (n = 20)	4.25 ± 1.85 (n = 174)	<0.001
IBIL (μmol/L)	7.98 ± 7.87 (n = 20)	8.83 ± 4.02 (n = 174)	0.393
TBIL (μmol/L)	15.08 ± 24.8 (n = 20)	13.16 ± 5.62 (n = 174)	0.001
RBC (10^12^/L)	4.41 ± 0.8 (n = 20)	4.32 ± 0.62 (n = 164)	0.242
HGB (g/L)	130.05 ± 24.19 (n = 20)	128.06 ± 18.22 (n = 164)	0.222
WBC (10^9^/L)	7.58 ± 3.22 (n = 20)	5.66 ± 1.69 (n = 164)	0.013
LY (10^9^/L)	2 ± 0.71 (n = 20)	1.74 ± 0.59 (n = 164)	0.142
PLT (10^9^/L)	256.6 ± 102.96 (n = 20)	224.36 ± 56.79 (n = 164)	0.021

Data presented as mean ± standard deviation, or n (%).

BMI (kg/m^2^), Body mass index; FBG (mmol/L), Fasting blood glucose; HbA1c (%), Glycated hemoglobin; FI (μU/mL), Fasting insulin; HOMA-IR, Homeostasis model assessment of insulin resistance; TC (mmol/L), Total cholesterol; TG (mmol/L), Triglyceride; LDL-c (mmol/L), Low-density lipoprotein cholesterol; HDL-c (mmol/L), High-density lipoprotein cholesterol; ApoB (g/L), Apolipoprotein B; non-HDL-c (mmol/L), Non-HDL cholesterol; LIP (mmol/L), Lipoprotein; SBP (mmHg), Systolic blood pressure; DBP (mmHg), Diastolic blood pressure; ALT (U/L), Alanine aminotransferase; AST (U/L), Aspartate aminotransferase; ALB (g/L), Serum albumin; TP (g/L), Total protein; A/G, Albumin/globulin; LAP (U/L), Leucine aminopeptidase; GT (U/L), glutamyl transpeptidase; GR (U/L), Glutathione Reductase; DBIL (μmol/L), Direct bilirubin; IBIL (μmol/L), Indirect bilirubin; TBIL (μmol/L), Total bilirubin; RBC (10^12^/L), Red blood cell; HGB (g/L), Hemoglobin; WBC (10^9^/L), White blood cell; LY (10^9^/L), Lymphocyte; PLT (10^9^/L), Platelet.

### Comparative analysis of each index after WMT treatment and baseline

**Figure 2:**
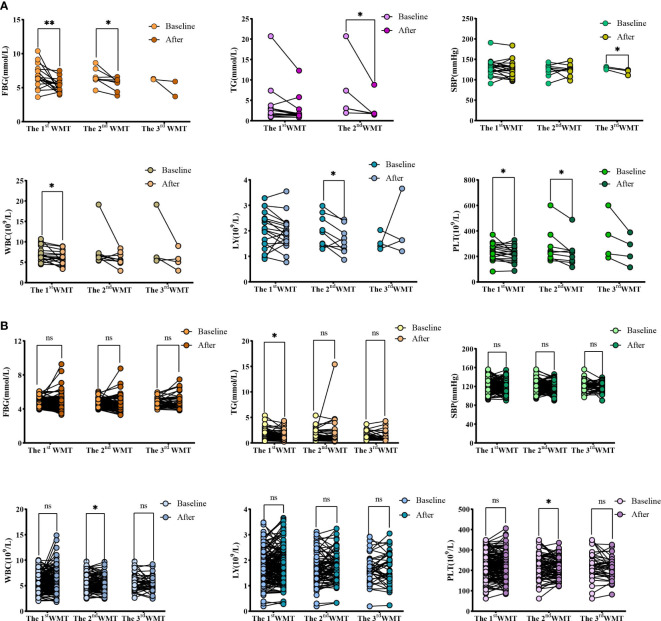
Changes of FBG, TG, SBP, WBC, LY and PLT levels after 1-3 times of washing and transplanting. **(A)** Changes of FBG, TG, SBP, WBC, LY and PLT in high blood glucose group; **(B)** Changes of FBG, TG, SBP, WBC, LY and PLT in normal blood glucose group. FBG, Fasting blood glucose; TG, Triglyceride; SBP, Systolic blood pressure; WBC, White blood cell; LY, Lymphocyte; PLT, Platelet. * indicates p < 0.05 ** indicates p < 0.01; ns, not significant.

**Table 3** and **Figure 2** show the effects of WMT on blood glucose, blood lipids, blood pressure, liver function and blood routine in patients with abnormal blood glucose metabolism. The results showed that WMT had a significant reducing effect on FBG in the short term (from 6.70 ± 1.65 to 5.29 ± 0.99mmol/L, p = 0.006) and medium term (from 6.58 ± 1.20 to 5.56 ± 0.95mmol/L, p = 0.025) in the high blood glucose group (p < 0.05), and also showed a decreasing effect in the long term (from 6.27 ± 0.08 to 4.80 ± 1.56mmol/L), but because the number of people was too small, it was not significant in the long term (p = 0.425). In the high blood glucose group, HbA1c decreased from 7.05 ± 0.49 to 6.95 ± 0.21mmol/L in the short term. FI decreased from 15.77 ± 10.3 to 11.88 ± 5.78 μU/mL in the short term and from 13.91 ± 11.8 to 11.98 ± 2.48 μU/mL in the long term. The HOMA-IR decreased from 4.73 ± 3.27 to 2.54 ± 1.36 in the short term and from 3.85 ± 3.25 to 2.64 ± 1.36 in the long term, but it was not significant. Overall, WMT has a good effect on improving blood glucose metabolism in patients with abnormal blood glucose metabolism.

**Table 3:** The comparison values of each index in high blood glucose group and normal blood glucose group in the short term, medium term and long term with baseline during the treatment of washed microbiota transplantation.

Items	Baseline	Short term	p-Value	Baseline	Medium term	p-Value	Baseline	Long term	p-Value
**High blood glucose group**									
BMI (kg/m^2^)	24.75 ± 5.56 (n = 17)	24.32 ± 5.37 (n = 17)	0.303	23.54 ± 4.12 (n = 7)	22.62 ± 4.26 (n = 7)	0.067	21.11 ± 5.38 (n = 2)	19.85 ± 3.25 (n = 2)	0.557
FBG (mmol/L)	6.70 ± 1.65 (n = 18)	5.29 ± 0.99 (n = 18)	0.006	6.58 ± 1.20 (n = 8)	5.56 ± 0.95 (n = 8)	0.025	6.27 ± 0.08 (n = 2)	4.80 ± 1.56 (n = 2)	0.425
HbA1c (%)	7.05 ± 0.49 (n = 2)	6.95 ± 0.21 (n = 2)	0.874	/	/	/	/	/	/
FI (μU/mL)	15.77 ± 10.36 (n = 7)	11.88 ± 5.78 (n = 7)	0.359	/	/	/	13.91 ± 11.85 (n = 2)	11.98 ± 2.48 (n = 2)	0.820
HOMA-IR	4.73 ± 3.27 (n = 7)	2.54 ± 1.36 (n = 7)	0.134	/	/	/	3.85 ± 3.25 (n = 2)	2.64 ± 1.36 (n = 2)	0.532
TC (mmol/L)	5.36 ± 1.56 (n = 14)	4.98 ± 1.09 (n = 14)	0.338	6.68 ± 1.87 (n = 5)	5.24 ± 1.09 (n = 5)	0.136	/	/	/
TG (mmol/L)	3.78 ± 5.16 (n = 14)	2.62 ± 3.03 (n = 14)	0.198	7.22 ± 7.83 (n = 5)	3.09 ± 3.21 (n = 5)	0.043	/	/	/
LDL-c (mmol/L)	2.81 ± 1.00 (n = 14)	2.86 ± 1.13 (n = 14)	0.860	3.16 ± 1.29 (n = 5)	2.78 ± 1.26 (n = 5)	0.366	/	/	/
HDL-c (mmol/L)	1.06 ± 0.29 (n = 14)	1.09 ± 0.27 (n = 14)	0.575	0.83 ± 0.21 (n = 5)	1.06 ± 0.21 (n = 5)	0.082	/	/	/
ApoB (g/L)	1.07 ± 0.32 (n = 14)	1.03 ± 0.20 (n = 14)	0.676	1.28 ± 0.41 (n = 5)	1.04 ± 0.15 (n = 5)	0.314	/	/	/
non-HDL-c (mmol/L)	4.30 ± 1.75 (n = 14)	3.90 ± 1.07 (n = 14)	0.327	5.85 ± 2.05 (n = 5)	4.18 ± 1.21 (n = 5)	0.103	/	/	/
LIP (mmol/L)	198.64 ± 228.59 (n = 5)	228.54 ± 250.33 (n = 5)	0.168	/	/	/	/	/	/
SBP (mmHg)	130.20 ± 19.87 (n = 20)	127.00 ± 20.83 (n = 20)	0.315	124.11 ± 15.66 (n = 9)	124.22 ± 14.47 (n = 9)	0.984	128.67 ± 3.51 (n = 3)	119.00 ± 7.00 (n = 3)	0.048
DBP (mmHg)	78.75 ± 10.84 (n = 20)	76.30 ± 11.47 (n = 20)	0.275	75.22 ± 10.98 (n = 9)	79.78 ± 9.50 (n = 9)	0.243	75.33 ± 0.58 (n = 3)	73.00 ± 5.20 (n = 3)	0.556
ALT (U/L)	23.84 ± 13.43 (n = 19)	24.21 ± 13.96 (n = 19)	0.907	30.25 ± 13.66 (n = 8)	22.75 ± 14.5 (n = 8)	0.266	32.25 ± 13.15 (n = 4)	23.00 ± 9.70 (n = 4)	0.334
AST (U/L)	26.42 ± 11.75 (n = 19)	26.84 ± 12.97 (n = 19)	0.884	31.13 ± 13.44 (n = 8)	24.00 ± 9.93 (n = 8)	0.217	37.25 ± 17.23 (n = 4)	36.75 ± 20.97 (n = 4)	0.938
ALB (g/L)	38.00 ± 6.46 (n = 19)	39.03 ± 5.54 (n = 19)	0.455	36.08 ± 9.11 (n = 8)	39.27 ± 8.03 (n = 8)	0.220	32.33 ± 14.35 (n = 3)	38.13 ± 13.11 (n = 3)	0.058
TP (g/L)	67.94 ± 5.23 (n = 19)	67.92 ± 5.58 (n = 19)	0.991	65.41 ± 4.09 (n = 8)	67.74 ± 6.93 (n = 8)	0.385	62.17 ± 5.51 (n = 3)	73.80 ± 8.25 (n = 3)	0.121
A/G	1.33 ± 0.37 (n = 19)	1.42 ± 0.39 (n = 19)	0.323	1.34 ± 0.54 (n = 8)	1.49 ± 0.53 (n = 8)	0.435	1.26 ± 0.82 (n = 3)	1.31 ± 0.85 (n = 3)	0.586
LAP (U/L)	32.84 ± 9.27 (n = 11)	33.16 ± 8.21 (n = 11)	0.848	35.82 ± 11.72 (n = 5)	36.68 ± 10.63 (n = 5)	0.828	46.05 ± 13.93 (n = 2)	49.35 ± 6.29 (n = 2)	0.856
GT (U/L)	58.27 ± 84.05 (n = 10)	47.62 ± 53.10 (n = 10)	0.316	75.62 ± 119.66 (n = 5)	54.08 ± 71.22 (n = 5)	0.379	149.00 ± 197.99 (n = 2)	102.70 ± 133.93 (n = 2)	0.493
GR (U/L)	61.08 ± 15.92 (n = 16)	59.23 ± 12.02 (n = 16)	0.610	65.03 ± 18.02 (n = 6)	56.18 ± 10.62 (n = 6)	0.230	59.00 ± 31.11 (n = 2)	65.50 ± 0.71 (n = 2)	0.821
DBIL (μmol/L)	6.01 ± 10.66 (n = 19)	5.87 ± 9.35 (n = 19)	0.801	10.00 ± 16.08 (n = 8)	6.96 ± 10.58 (n = 8)	0.191	18.23 ± 27.08 (n = 3)	12.72 ± 15.57 (n = 3)	0.494
IBIL (μmol/L)	8.28 ± 7.97 (n = 19)	8.22 ± 5.70 (n = 19)	0.952	11.14 ± 11.98 (n = 8)	11.43 ± 13.10 (n = 8)	0.828	16.93 ± 20.45 (n = 3)	14.75 ± 9.63 (n = 3)	0.760
TBIL (μmol/L)	15.54 ± 25.39 (n = 19)	14.09 ± 14.67 (n = 19)	0.593	24.11 ± 38.83 (n = 8)	18.39 ± 23.64 (n = 8)	0.336	45.87 ± 64.33 (n = 3)	19.83 ± 11.95 (n = 3)	0.480
RBC (10^12^/L)	4.43 ± 0.82 (n = 19)	4.38 ± 0.77 (n = 19)	0.575	4.17 ± 1.03 (n = 9)	4.52 ± 0.73 (n = 9)	0.070	3.53 ± 1.30 (n = 4)	4.40 ± 1.10 (n = 4)	0.077
HGB (g/L)	132.42 ± 22.34 (n = 19)	130.84 ± 16.43 (n = 19)	0.577	123.44 ± 33.19 (n = 9)	131.56 ± 23.08 (n = 9)	0.203	103.00 ± 42.65 (n = 4)	122.50 ± 32.28 (n = 4)	0.246
WBC (10^9^/L)	6.97 ± 1.76 (n = 19)	6.04 ± 1.58 (n = 19)	0.016	7.70 ± 4.33 (n = 9)	5.77 ± 1.60 (n = 9)	0.151	9.21 ± 6.63 (n = 4)	5.7 ± 2.51 (n = 4)	0.230
LY (10^9^/L)	2.03 ± 0.71 (n = 19)	1.93 ± 0.63 (n = 19)	0.358	1.98 ± 0.62 (n = 9)	1.63 ± 0.53 (n = 9)	0.019	1.56 ± 0.33 (n = 4)	2.03 ± 1.10 (n = 4)	0.498
PLT (10^9^/L)	238.47 ± 65.23 (n = 19)	217.11 ± 58.10 (n = 19)	0.037	275.89 ± 136.38 (n = 9)	230.11 ± 106.85 (n = 9)	0.031	346.00 ± 187.35 (n = 4)	249.75 ± 118.42 (n = 4)	0.099
**Normal blood glucose group**									
BMI (kg/m^2^)	22.09 ± 3.94 (n = 172)	21.99 ± 3.89(n = 172)	0.386	21.7 ± 3.67(n = 96)	21.51 ± 3.7 (n = 96)	0.375	22.07 ± 3.99 (n = 45)	21.98 ± 4.04(n = 45)	0.787
FBG (mmol/L)	4.63 ± 0.48 (n = 154)	4.59 ± 0.78 (n = 154)	0.538	4.63 ± 0.5 (n = 86)	4.54 ± 0.82(n = 86)	0.261	4.77 ± 0.55 (n = 42)	4.79 ± 0.83 (n = 42)	0.893
HbA1c (%)	5.15 ± 0.50 (n = 2)	5.20 ± 0.71 (n = 2)	0.795	/	/	/	/	/	/
FI (μU/mL)	8.52 ± 5.31 (n=53)	8.80 ± 5.46 (n=53)	0.624	8.98 ± 5.48 (n=29)	8.95 ± 5.8 (n=29)	0.960	11.49 ± 6.36 (n=15)	10.97 ± 6.7 (n=15)	0.667
HOMA-IR	1.77 ± 1.16 (n=53)	1.87 ± 1.45 (n=53)	0.489	1.90 ± 1.28 (n=29)	1.95 ± 1.44 (n=29)	0.729	2.46 ± 1.5 (n=15)	2.33 ± 1.57 (n=15)	0.615
TC (mmol/L)	4.76 ± 1.06 (n=110)	4.66 ± 1.03 (n=110)	0.206	4.72 ± 1.06 (n=63)	4.58 ± 0.93 (n=63)	0.197	4.94 ± 1.12 (n=29)	4.61 ± 1 (n=29)	0.039
TG (mmol/L)	1.31 ± 0.84 (n=110)	1.19 ± 0.69 (n=110)	0.030	1.33 ± 0.9 (n=63)	1.5 ± 2.03 (n=63)	0.449	1.42 ± 0.79 (n=29)	1.29 ± 0.96 (n=29)	0.259
LDL-c (mmol/L)	2.88 ± 0.93 (n=110)	2.82 ± 0.89 (n=110)	0.402	2.79 ± 0.9 (n=63)	2.64 ± 0.82 (n=63)	0.124	2.92 ± 0.99 (n=29)	2.69 ± 0.88 (n=29)	0.099
HDL-c (mmol/L)	1.3 ± 0.33 (n=110)	1.31 ± 0.31 (n=110)	0.631	1.35 ± 0.34 (n=63)	1.32 ± 0.3 (n=63)	0.456	1.38 ± 0.31 (n=29)	1.34 ± 0.31 (n=29)	0.150
ApoB (g/L)	0.92 ± 0.24 (n=110)	0.91 ± 0.24 (n=110)	0.412	0.91 ± 0.23 (n=63)	0.90 ± 0.25 (n=63)	0.705	0.96 ± 0.21 (n=29)	0.96 ± 0.24 (n=29)	0.796
non-HDL-c (mmol/L)	3.46 ± 1.01 (n=110)	3.35 ± 0.98 (n=110)	0.148	3.38 ± 0.97 (n=63)	3.25 ± 0.87 (n=63)	0.225	3.57 ± 0.99 (n=29)	3.28 ± 0.92 (n=29)	0.049
LIP (mmol/L)	156.14 ± 303.68 (n=23)	156.29 ± 278.95 (n=23)	0.986	77.49 ± 75.35 (n=10)	83.04 ± 78.93 (n=10)	0.444	60.35 ± 24.68 (n=2)	42.60 ± 2.12 (n=2)	0.521
SBP (mmHg)	121.26 ± 12.75 (n=174)	120.38 ± 11.79 (n=174)	0.389	120.16 ± 12.85 (n=98)	119.6 ± 11.94 (n=98)	0.708	120.87 ± 12.11 (n=45)	119.49 ± 10.09 (n=45)	0.499
DBP (mmHg)	76.91 ± 9.38 (n=174)	76.44 ± 9.12 (n=174)	0.566	76.38 ± 9.47 (n=98)	74.86 ± 8.31 (n=98)	0.168	76.80 ± 9.41 (n=45)	77.16 ± 8.03 (n=45)	0.823
ALT (U/L)	20.65 ± 16.8 (n=168)	19.55 ± 13.2 (n=168)	0.355	20.25 ± 15.25 (n=88)	20.5 ± 13.32 (n=88)	0.863	19.91 ± 11.46 (n=43)	21.12 ± 18.55 (n=43)	0.633
AST (U/L)	21.04 ± 7.22 (n=168)	21.29 ± 7.59 (n=168)	0.671	20.78 ± 6.35 (n=88)	21.80 ± 7.36 (n=88)	0.201	21.26 ± 6.09 (n=43)	23.33 ± 12.34 (n=43)	0.177
ALB (g/L)	40.71 ± 4.36 (n=168)	40.53 ± 3.88 (n=168)	0.562	40.93 ± 4.77 (n=88)	40.36 ± 4.06 (n=88)	0.205	40.52 ± 5.01 (n=43)	40.4 ± 4.18 (n=43)	0.880
TP (g/L)	68.34 ± 5.99 (n=168)	67.79 ± 5.75 (n=168)	0.234	68.41 ± 6.44 (n=88)	67.63 ± 5.67 (n=88)	0.231	68.65 ± 5.97 (n=43)	67.64 ± 5.48 (n=43)	0.226
A/G	1.53 ± 0.33 (n=168)	1.53 ± 0.28 (n=168)	0.995	1.56 ± 0.38 (n=88)	1.52 ± 0.3 (n=88)	0.368	1.51 ± 0.41 (n=43)	1.52 ± 0.32 (n=43)	0.869
LAP (U/L)	29.71 ± 5 (n=112)	30.25 ± 5.01 (n=112)	0.047	29.36 ± 4.86 (n=63)	29.69 ± 5.57 (n=63)	0.340	29.41 ± 5.45 (n=31)	29.62 ± 5.14 (n=31)	0.672
GT (U/L)	24.17 ± 14.4 (n=121)	24.33 ± 14.97 (n=121)	0.789	24.98 ± 15.38 (n=70)	24.77 ± 16.11 (n=70)	0.838	27.85 ± 14.00 (n=35)	26.35 ± 12.68 (n=35)	0.318
GR (U/L)	56.34 ± 12.92 (n=119)	56.96 ± 13.46 (n=119)	0.340	54.24 ± 10.62 (n=62)	54.65 ± 11.74 (n=62)	0.682	53.3 ± 11.8 (n=28)	53.47 ± 13.1 (n=28)	0.912
DBIL (μmol/L)	4.19 ± 1.79 (n = 168)	4.16 ± 2.04 (n = 168)	0.761	4.13 ± 1.71 (n = 88)	4.08 ± 1.87 (n = 88)	0.769	4.09 ± 1.71 (n = 43)	4.15 ± 1.57 (n = 43)	0.826
IBIL (μmol/L)	8.72 ± 3.77 (n = 168)	8.47 ± 3.97 (n = 168)	0.336	8.75 ± 3.79 (n = 88)	8.42 ± 3.95 (n = 88)	0.326	9.19 ± 4.07 (n = 43)	8.96 ± 3.91 (n = 43)	0.694
TBIL (μmol/L)	13.01 ± 5.3 (n = 168)	12.64 ± 5.8 (n = 168)	0.287	12.94 ± 5.26 (n = 88)	12.51 ± 5.62 (n = 88)	0.357	13.29 ± 5.62 (n = 43)	13.10 ± 5.32 (n = 43)	0.819
RBC (10^12^/L)	4.32 ± 0.62 (n = 164)	4.29 ± 0.62 (n = 164)	0.143	4.32 ± 0.54 (n = 85)	4.31 ± 0.56 (n = 85)	0.721	4.31 ± 0.56 (n = 39)	4.34 ± 0.55 (n = 39)	0.628
HGB (g/L)	128.06 ± 18.22 (n = 164)	127.06 ± 18.19 (n = 164)	0.140	127.21 ± 19.15 (n = 85)	127.46 ± 16.85 (n = 85)	0.843	127.23 ± 21.56 (n = 39)	128.49 ± 18.46 (n = 39)	0.476
WBC (10^9^/L)	5.66 ± 1.69 (n = 164)	5.84 ± 2.12 (n = 164)	0.178	5.60 ± 1.64 (n = 85)	5.31 ± 1.58 (n = 85)	0.021	5.62 ± 1.64 (n = 39)	5.34 ± 1.47 (n = 39)	0.209
LY (10^9^/L)	1.74 ± 0.59 (n = 164)	1.77 ± 0.66 (n = 164)	0.392	1.69 ± 0.58 (n = 85)	1.74 ± 0.57 (n=85)	0.337	1.66 ± 0.54 (n = 39)	1.68 ± 0.64 (n = 39)	0.794
PLT (10^9^/L)	224.36 ± 56.79 (n=164)	222.84 ± 58.66 (n=164)	0.536	220.73 ± 57.45 (n=85)	212.55 ± 46.86 (n=85)	0.042	223.56 ± 64.26 (n = 39)	211.77 ± 52.94 (n = 39)	0.111

Data presented as mean ± standard deviation, or n (%).

At the same time, in the high blood glucose group, WMT significantly reduced TG (from 7.22 ± 7.83 to 3.09 ± 3.21mmol/L, p = 0.043) in the mid-term (p < 0.05), indicating that WMT has the effect of reducing blood lipids. There was a significant long term reduction (p < 0.05) on SBP (from 128.67 ± 3.51 to 119.00 ± 7.00mmHg, p = 0.048), indicating that WMT has a blood pressure lowering effect. There was a significant short term reduction (p < 0.05) on WBC (from 6.97 ± 1.76 to 6.04 ± 1.58 109/L, p = 0.016), indicating that WMT may have the function of inhibiting inflammatory response. Significantly decreased LY (from 1.98 ± 0.62 to 1.63 ± 0.53 109/L, p = 0.019) in the medium term (p < 0.05), indicating that WMT may have an immunomodulatory effect. There was a significant reduction (p < 0.05) in the short term (from 238.47 ± 65.23 to 217.11 ± 58.10 109/L, p = 0.037) and mid-term (from 275.89 ± 136.38 to 230.11 ± 106.85 109/L, p = 0.031) of PLT, indicating that WMT may have a role in regulating cardiovascular disease. There was no significant change in FBG, HbA1c, FI, HOMA-IR, etc. in the short term, medium term and long term in the normal blood glucose group by WMT, that is, WMT had no effect on normoglycemic patients. But for TC (p = 0.039) and TG (p = 0.030) were significantly decreased (p < 0.05). Both the high blood glucose group and the normal blood glucose group had a decreasing trend in BMI before and after treatment, but there was no significant change.

### Correlation analysis of WMT on indicators affecting blood glucose regulation

**Figure 3:**
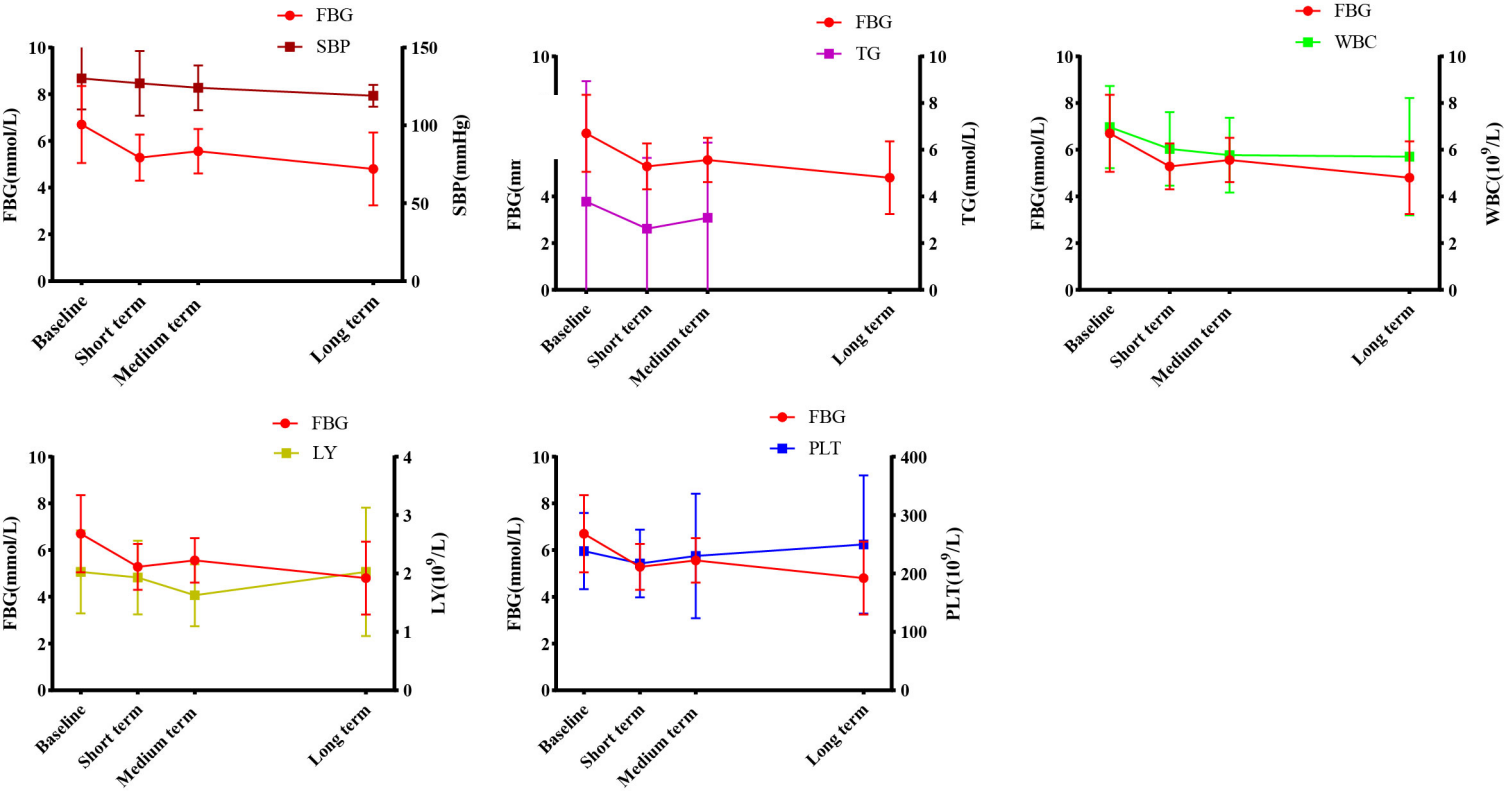
Correlation analysis of WMT on blood glucose regulation. FBG, Fasting blood glucose; TG, Triglyceride; SBP, Systolic blood pressure; WBC, White blood cell; LY, Lymphocyte; PLT, Platelet.

Earlier we found that during the treatment process, WMT had a significant improvement effect on FBG, TG, SBP, WBC, LY and PLT in the high blood glucose group. In order to find the relevant factors that affect the regulation of blood glucose by WMT, correlation analysis was carried out on the above-mentioned indicators with significant regulation effect. As shown in **Figure 3**, we found that in high blood glucose levels, FBG was positively correlated with TG (r=0.973), SBP (r=0.866), WBC (r=0.926), and LY (r=0.089); FBG was negatively correlated with PLT (r=-0.069). Our data show that during WMT treatment, blood pressure, blood lipids, anti-inflammatory response and immune function are simultaneously affected while improving blood glucose, and there is an interaction link. This provides us with a good treatment idea for the treatment of patients with abnormal blood glucose metabolism, that is, in addition to lowering blood glucose, it can also assist in the treatment of blood pressure and blood lipids, inhibit inflammation and improve immunity while treating high blood glucose.

### Evaluation of WMT clinical therapeutic effect on blood glucose levels

All the enrolled patients were divided into high blood glucose group and normal blood glucose group according to blood glucose baseline. Patients were regrouped according to changes in blood glucose levels after 1-3 WMT treatments (**Table 4**). There were significant changes in blood glucose levels in the short term in patients with high blood glucose group. In the high blood glucose group, 72.22% returned to normal in the short term (p < 0.001), 57.14% returned to normal in the medium term (p = 0.076), and long term returned to normal is not significant (p = 0.317). Because the number of people is too small, there is no significant difference in the long term. Our data suggest that WMT can significantly change higher blood glucose levels in patients to normal levels in short term treatment; however, the efficacy of WMT remains to be explored. Our data show that WMT may significantly improve blood glucose levels after short term treatment, whereas blood glucose levels stabilize after medium and long term treatment.

**Table 4:** Comprehensive clinical efficacy of short, medium and long term treatment on blood glucose levels.

Data periods	Before therapy (n)	Therapeutic effect base on blood glucose levels			
		Unchanged group (n)	Changed group (n, %)	X^2^	p-Value
**High blood glucose group**					
Short term	18	5	13 (72.22%)	17.338	<0.001
Medium term	7	3	4 (57.14%)	3.150	0.076
Long term	2	0	2 (100%)	1.000	0.317
**Normal blood glucose group**					
Short term	154	149	5(3.36%)	3.253	0.071
Medium term	86	83	3 (4.65%)	1.375	0.244
Long term	42	37	5 (13.51%)	3.403	0.065

The definition of unchanged and changed of high blood glucose group were still high blood glucose group and changed to normal.

The definition of unchanged and changed of normal blood glucose were still normal and changed to high blood glucose group.

### Evaluation of the therapeutic effect of WMT on the ASCVD risk

According to ASCVD risk stratification, patients were divided into extremely high risk group, high risk group, medium risk group and low risk group. After WMT treatment, patients were regrouped into no risk changed group and the risk-changed group (**Table 5**). Acute coronary syndrome, coronary heart disease, stroke, and peripheral atherosclerosis were included in the extremely high risk group, and these patients were not reassigned after WMT and were not listed in **Table 5**.

**Table 5:** Effects of short, medium, and long term treatment on atherosclerotic cardiovascular disease risk classification.

Data periods	Before therapy (n)	Therapeutic effect base on ASCVD risk stratification			
		Unchangedgroup (n)	Changedgroup (n, %)	X^2^	p-Value
**High risk group**					
Short term	24	18	6(25%)	4.792	0.029
Medium term	15	10	5(33.3%)	3.896	0.050
Long term	7	6	1(14.3%)	1.077	0.299
**Medium risk group**					
Short term	10	8	2(20%)	0.556	0.456
Medium term	6	5	1(16.7%)	1.091	0.296
Long term	5	4	1(20%)	1.111	0.292
**Low risk group**					
Short term	84	79	5(5.8%)	3.298	0.069
Medium term	44	43	1(2.3%)	1.011	0.315
Long term	19	17	2(10.5%)	0.528	0.468

The definition of unchanged of high-, medium-, and low-risk groups were still high-, medium-, and low-risk after WMT procedures, respectively.

In the high-risk group, the effects of short term and medium term WMT treatment were significant. Short term 25% and medium term 33.3% were classified as medium risk group or below (p < 0.05). In the medium risk group, the effects of short, medium and long term WMT treatment were not significant, but the number of people was reduced. In the ASCVD low risk group, this change was not statistically significant, suggesting that WMT does not increase the risk of ASCVD.

## Discussion

To the best of our knowledge, this is one of the few clinical study to investigate the effects of WMT on patients with abnormal blood glucose metabolism, especially those with high blood glucose, in South China, indicating that regulating gut microbiota may be a new method for the treatment of abnormal blood glucose metabolism. These data show that WMT has a significant short and medium term effect on improving blood glucose in patients with high blood glucose, and at the same time, it has significant hypolipidemic and blood pressure-lowering effects on blood lipids and blood pressure in patients with high blood glucose. Overall, WMT significantly reduced the risk of high risk ASCVD in the short and medium term, and there was no significant difference between the medium and low risk groups, suggesting that WMT could reduce the short and medium term risk of ASCVD without increasing the risk of ASCVD.

Numerous studies have shown that gut microbiota disturbances may influence the progression of diabetes (32, 33). Patients with T2DM have a moderate dysbiosis between butyrate-producing and lactic acid bacteria (34). Furthermore, various metabolites, such as short chain fatty acids (SCFAs), produced by the gut microbiota differ significantly between T2DM and normal hosts (35). Studies have shown that ingestion of probiotics can effectively improve gut microbiota disturbances and relieve symptoms in diabetic patients, such as remodeling the formation of gut microbiota to improve or control the disease state (36). FMT is an approach to treat disease by rebuilding the microbiota (37). There is growing evidence that the therapeutic potential of FMT is based on an established clinical program that has become the first-line treatment for recurrent *Clostridium difficile* infections (38). However, understanding the impact of gut microbiota on metabolic disease is still in its infancy, and data on the effect of FMT on T2DM are still scarce. In our study, WMT had a significant short and medium term improvement in blood glucose in patients with high blood glucose. It is possible to restore gut microbiota balance to promote host homeostasis (39).

The potential effects of FMT on glucose homeostasis and insulin sensitivity in humans have been identified. FMT using lean donors was sufficient to improve glucose homeostasis in obese individuals, with a small decrease in HbA1c 6 weeks after FMT, which was associated with changes in gut microbiota (40). Another FMT double-blind placebo-controlled trial reported a slight improvement in HbA1c at 12 weeks (41). Our results for FBG and HbA1c showed a decreasing trend, providing further evidence for the hypoglycemic effect of WMT. As reported in the literature, FMT can show a restored phenotype following transfer of the donor’s gut microbiota to the recipient (42). T2DM mice had reduced FI levels and improved HOMA-IR after reconstitution of their microbiota from normal mouse feces (17). Likewise, in a clinical trial, obese patients treated with FMT in lean healthy individuals showed positive changes in homeostasis model assessment of insulin sensitivity (HOMA-IS) (43). Our study did not observe a significant improvement in FI and HOMA-IR after WMT, although some studies showed that FMT significantly improved HOMA-IS (44). Our non-significant results may be due to the small sample size in our study, the lack of HOMA-IR indicators in many of our high blood glucose patients, and due to technical factors including the accuracy of the instrument, among others. For the current study, gut dysbiosis was associated with the development of insulin resistance and diabetes (42). The mechanism of gut microbiota-induced improvement in insulin resistance may be through altering body energy balance or reducing obesity induced by a high-fat diet. However, the exact mechanism needs to be fully explored.

FMT is associated with low-grade inflammation characterized by metabolic disturbances. Therapeutic FMT has been reported to reduce the secretion of inflammatory factors and trigger multiple immune-mediated signaling pathways in colitis (45). As mentioned in one study, transplantation of gut microbiota such as *Faecalibacterium prausnitzii* prevented inflammatory damage to the pancreas (46). These findings are consistent with our results, the WBC in the high blood glucose group after WMT showed a decreasing trend in the short, medium and long term, and there were significant differences in the short term, indicating that WMT may inhibit the inflammatory response. LY showed a significant downward trend in the medium term, indicating that WMT may have an immunomodulatory effect. IL-6 and TNF-α are pro-inflammatory cytokines with multiple functions that can directly act on islet cells and cause islet β-cell damage (47). Low-grade chronic inflammation caused by microbiota imbalance usually leads to islet structural damage and impaired islet β-cell function, and islet β-cell apoptosis is the underlying cause of islet structural damage. Therefore, FMT treatment of inflammatory response and apoptosis of islet β cells can reverse islet damage and dysfunction, which may be a method worth exploring.

T2DM patients often have one or more components of metabolic syndrome, such as hypertension, dyslipidemia, obesity, etc., which significantly increase the risk, progression speed and harm of T2DM complications. Comprehensive treatment should be based on the control of blood glucose, blood pressure, blood lipids and body weight, antiplatelet therapy and other lifestyle improvements. In our study, in addition to improving blood glucose, WMT has blood lipid-lowering and blood pressure-lowering effects in patients with high blood glucose, and has a significant reduction in platelets in the short and medium term, which is in line with the recommendations of antiplatelet therapy. We speculate that the improvement of abnormal blood glucose metabolism after WMT is due to the improvement of gut microbiota after WMT, which comprehensively regulates blood lipids, blood pressure, and platelets. The short term goal of diabetes treatment is to eliminate diabetes symptoms and prevent acute complications by controlling hyperglycemia and metabolic disorders. The long term goal of diabetes treatment is to prevent chronic complications, improve patients’ quality of life and prolong life through good metabolic control. A scientific and reasonable T2DM treatment strategy should be comprehensive, including control of blood glucose, blood pressure, blood lipids, and body weight, antiplatelet therapy, and lifestyle improvements. Although there are several studies on WMT function, such as Liang et al. showed that WMT treatment can alter blood lipids in patients with hyperlipidemia and hypolipidemia without serious adverse events (48). Studies by Zhong et al. have shown that WMT has antihypertensive effects in hypertensive patients (49). Pan et al. showed that WMT significantly improved children with autism spectrum disorder, gastrointestinal symptoms and sleep disturbance, and reduced systemic inflammation (50). The mechanism of FMT-induced disease remission remains largely unexplained. Diabetic symptoms may be alleviated by synergistic effects between gut commensal microbiota after FMT treatment, or may trigger multiple immune-inflammatory processes and pathways.

Future research should focus on the bacterial species and functional changes associated with FMT treatment in diabetic patients and how FMT affects the metabolism of other organs in long term improvement. Due to the complexity of the gut microbiota, further studies should explore whether specific microbial species or communities in FMT are dedicated to the prevention and treatment of diabetes. This may provide a new perspective and reference. Many factors influence the outcome of FMT, namely donor selection and preparation, sample handling, mode of administration, and colonization resistance (51). FMT-related AEs are a challenge for FMT applications. In most cases, mild gastrointestinal AEs were well tolerated by FMT (52). Our WMT project based on Zhang’s criteria found no serious AEs during and after WMT. Zhang et al. prepared washed microbial flora by repeating centrifugation and suspension three times on the basis of an automatic purification system, which significantly reduced AEs (25). At present, the understanding of the effect of WMT on the gut microbiota on metabolic diseases is still in its infancy, and data on the effect of WMT on abnormal blood glucose metabolism are still lacking. This is the first large-scale retrospective trial of abnormal blood glucose metabolism in China, including high blood glucose group and normal blood glucose group. The clinical evidence of the effect of WMT on blood glucose metabolism has been established, which lays the foundation for subsequent studies on the effects of gut microbiota (53), metabolic markers (54), and diet (55) regulation on abnormal blood glucose metabolism. Taken together, these results suggest that restoring gut microbiota may serve as a promising treatment for abnormal blood glucose metabolism; however, the mechanism of action requires further investigation.

This study has several limitations. First, this study mainly focused on the analysis of clinical blood glucose metabolism, blood lipid metabolism, and liver function, etc. The gut microbiota metagenomics and metabolomics before and after WMT have not been evaluated. Therefore, the lowering effect of WMT on blood glucose and related microbiota is unclear. Second, the impact of patient compliance. A small number of patients did not undergo long term treatment after short and medium term treatment, and returned to the hospital at short or long intervals for evaluation of the long term benefit of WMT treatment. Therefore, more data are needed to confirm the long term efficacy of WMT in the treatment of abnormal blood glucose metabolism. Third, we did not consider potential confounding factors between the main symptoms of WMT treatment and glycemia. Although data show that WMT can improve abnormal blood glucose metabolism in the short and medium term, we need large-scale prospective studies to further validate our conclusions.In the future, we plan to conduct a large-sample prospective study to verify the effect of WMT on blood glucose metabolism.

## Conclusion

WMT has a significant effect of improving blood glucose, and at the same time, it has a significant effect on reducing blood lipid and blood pressure in patients with high blood glucose. WMT reduces the short- and medium-term risk of ASCVD without increasing the long term risk of ASCVD. Therefore, the regulation of gut microbiota by WMT may provide a new clinical approach for the treatment of abnormal blood glucose metabolism.

## Data availability statement

The original contributions presented in the study are included in the article/supplementary material. Further inquiries can be directed to the corresponding authors.

## Ethics statement

The studies involving human participants were reviewed and approved by the Ethics Committee (No. 2017-98) in accordance with the Declaration of Helsinki at the First Affiliated Hospital of Guangdong Pharmaceutical University, Guangzhou, China. The patients/participants provided their written informed consent to participate in this study.

## Author contributions

X-XH, Q-PW, and LW designed the concept of the study. M-QL, Y-TX, X-JL, QZ, W-YL, and J-TX collected and analyzed the data. TL and W-YL were the statistics consultant, QZ was the consultant for endocrinology. LW wrote the draft manuscript. All authors contributed to the article and approved the submitted version.

## Funding

This study was supported by the Key Technologies R&D Program of Guangdong Province (2022B1111070006), the Natural Science Foundation of Guangdong Province (2019A1515010125), and the Department of Education of Guangdong Province (2021KCXTD025).

## Acknowledgments

We sincerely thank all patients in the study and all funding agencies that supported the study.

## Conflict of interest

The authors declare that the research was conducted in the absence of any commercial or financial relationships that could be construed as a potential conflict of interest.

## Publisher's note

All claims expressed in this article are solely those of the authors and do not necessarily represent those of their affiliated organizations, or those of the publisher, the editors and the reviewers. Any product that may be evaluated in this article, or claim that may be made by its manufacturer, is not guaranteed or endorsed by the publisher.
